# Characterisation of a Silicon Photomultiplier Based Oncological Brachytherapy Fibre Dosimeter

**DOI:** 10.3390/s24030910

**Published:** 2024-01-30

**Authors:** Massimo Caccia, Agnese Giaz, Marco Galoppo, Romualdo Santoro, Micheal Martyn, Carla Bianchi, Raffaele Novario, Peter Woulfe, Sinead O’Keeffe

**Affiliations:** 1 Dipartimento di Scienza e Alta Tecnologia, Università degli Studi dell’Insubria, via Valleggio 11, 22100 Como, Italy; agnese.giaz@uninsubria.it (A.G.); mgaloppo@studenti.uninsubria.it (M.G.); romualdo.santoro@uninsubria.it (R.S.); 2 Radiotherapy Department, Galway Clinic, Doughiska Road, H91 HHT0 Galway, Ireland; michael.martyn@galwayclinic.com; 3 Ospedale di Circolo di Varese, Università degli Studi dell’Insubria, Viale Borri, 57, 21100 Varese, Italy; carla.bianchi@asst-settelaghi.it (C.B.); raffaele.novario@gmail.com (R.N.); peter.wulfe@galwayclinic.com (P.W.); 4 Optical Fibre Sensors Research Centre, University of Limerick, V94 T9PX Limerick, Ireland; sinead.okeeffe@ul.ie

**Keywords:** brachytherapy, SiPM, HDR, LDR, dosimetry

## Abstract

Source localisation and real-time dose verification are at the forefront of medical research in brachytherapy, an oncological radiotherapy procedure based on radioactive sources implanted in the patient body. The ORIGIN project aims to respond to this medical community’s need by targeting the development of a multi-point dose mapping system based on fibre sensors integrating a small volume of scintillating material into the tip and interfaced with silicon photomultipliers operated in counting mode. In this paper, a novel method for the selection of the optimal silicon photomultipliers to be used is presented, as well as a laboratory characterisation based on dosimetric figures of merit. More specifically, a technique exploiting the optical cross-talk to maintain the detector linearity in high-rate conditions is demonstrated. Lastly, it is shown that the ORIGIN system complies with the TG43-U1 protocol in high and low dose rate pre-clinical trials with actual brachytherapy sources, an essential requirement for assessing the proposed system as a dosimeter and comparing the performance of the system prototype against the ORIGIN project specifications.

## 1. Introduction

Brachytherapy (BT) is a radiotherapy procedure during which radioactive sources, called seeds, are implanted into the patient’s body, in or close to the tumour [1,2,3,4]. According to the average activity of the source, clinical treatments are categorized as either Low Dose Rate (LDR, in the 0.4–2 Gy/h range) or High Dose Rate (HDR, in excess of 12 Gy/h) [5]. In LDR brachytherapy, the radioactive sources (e.g., ^125^I) are implanted permanently in the patient’s body, while in HDR brachytherapy, the seeds (e.g., ^192^Ir and ^60^Co) are only temporarily inserted. The main characteristics of the radioactive sources are reported in Table 1. The possibility of implementing treatments with a wide dose range and different radiation absorption lengths (Table 1) makes BT a reference procedure when treatment is required in the proximity of organs at risk (e.g., prostate and gynaecological cancer) [5,6].

Correct placement of the radiation source is vital to ensure the delivery of adequate radiation to the target area (tumour), while minimising exposure to nearby critical organs, such as, in the case of prostate and gynaecological cancers, the bladder, urethra and rectal wall. Current dosimetry techniques rely on pre- and post-treatment Computed Tomography (CT) and ultrasound imaging with dose calculated via computerised treatment planning system. Without direct in vivo monitoring of the dose delivered to the patient, there is no independent dose verification, with errors going undetected at the time of treatment. A 20 year systematic survey of endovaginal HDR-BT demonstrate acute endovaginal toxicity occurred in 20.6% of patients [7], while a Ireland-Northern Ireland study of over 3500 prostate cancer patients treated with LDR-BT showed that 58.5% of those survivors reported experiencing adverse physical symptoms, such as urinary incontinence, bowel problems, and erectile dysfunction [8,9,10,11,12]. The need to ensure accurate dose delivery through real-time dosimetry and source localisation was also demonstrated in a recent paper [13].

To date, real-time in vivo dosimetry is limited to a maximum of five points and the uncertainty associated with the sensor is 10% [14], limiting its use in the clinical workflow. There are currently no commercially available system for monitoring the radiation source location. As such, there is a clear and urgent need for a real-time in vivo dosimeter capable of monitoring the dose to the target area and organs at risk and also providing information of the source position during treatment.

ORIGIN (Optical fiber dose imaging for adaptive brachytherapy) is an international project supported by the European Commission (Grant agreement no. 871324-HORIZON2020 Framework Program) targeting the development of instruments and methods to provide real-time in vivo dose imaging and source localisation through a new optical fibre based sensor system that goes beyond the state of the art.

The specifications of the ORIGIN system are reported in Section 2. The principles and results from a laboratory characterisation aimed to identify the optimal sensors are described in Section 3. The commissioning and outcome of a pre-clinical study at hospital premises with brachytherapy HDR and LDR sources are given in Section 4, performed with the aim of assessing compliancy with the TG43-U1 protocol [15]. A critical analysis and an outlook concludes the paper.

**Table 1:** Main characteristics of the sources used for LDR-BT and HDR-BT. The reported absorption lengths refer to water. Data related to half-life and mean energy are from ref. [16,17]; absorptions are calculated according to the web application provided at https://web-docs.gsi.de/~stoe_exp/web_programs/x_ray_absorption/index.php (accessed on 31 July 2023) and verified according to the tables by the National Institute of Standard and technology, available at https://www.nist.gov/pml/x-ray-mass-attenuation-coefficients (accessed on 31 July 2023).

	^125^I	^192^Ir	^60^Co
BT-treatment kind	LDR-BT	HDR-BT	HDR-BT
Activity	∼15 MBq	∼100 GBq	∼100 GBq
Half-life	59.4 days	73.8 days	1925.3 days
Mean γ-ray energy	35.5 keV	380 keV	1250 keV
Abs. length (50%)	2.2 cm	6.4 cm	11 cm
Abs. length (90%)	7.5 cm	21 cm	37 cm

## 2. The ORIGIN System

The system under development is composed of sixteen Poly Methyl MethAcrylate (PMMA) optical fibres with a scintillating tip (sensor in the following), each connected to a Silicon PhotoMultiplier (SiPM). All SiPMs are read out with the WEEROC CITIROC1A ASIC [18] integrated into the CAEN DT5202 read-out board (https://www.caen.it/products/dt5202/, accessed on 31 July 2023). The basic features of the system are reported in Table 2.

The performance parameters are defined as follows:

- Dose sensitivity is the capability of the dosimeter to single out a dose signal from the background fluctuations at a given distance.
- Spatial resolution, ΔR(r), is the minimum displacement of the source at a selected distance (*r*), at any angle, for which the variation in the background-subtracted signal is three times its uncertainty.
- Statistical precision, SP(r;Δt), is the ratio between the counting uncertainty ($\sigma_{C}$) and the signal counts ($N_{Signal}$) for the selected measurement time window and at the chosen distance
  (1) $$SP\left(r;\Delta t\right)={\frac{\sigma_{C}}{N_{Signal}}}_{|r;\Delta t}.$$

At system design phase, the choice of the scintillating material for the sensor tip and of the light detector is of utmost importance. The scintillators chosen for the project are 1Y_2_O_3_:Eu + 4YVO_4_:Eu (YVO) and the Gd_2_O_2_S:Tb (Gadox), emitting at 619 nm and 544 nm respectively [19,20,21,22]. This choice is driven both by the emitted light wavelength and by the long scintillating decay time of the order of 500 μs [22]. The former is expected to limit the impact of the Cherenkov radiation emitted by the energy deposited in the clear fibre (the so-called stem effect). The latter enhances the signal as long as the system is operated in counting mode and single-photon sensitivity is provided, since a single gamma ray interaction effectively results in a train of single photons, with a multiplication factor on the signal. Hence, SiPMs are a natural choice for the detector since they provide single-photon sensitivity in a compact, low voltage, high photon detection efficiency and cost-effective device.

## 3. Laboratory Characterisation

Today, different types of SiPMs are produced by various companies, and the choice is driven by the specific application. In this study, a comparison is made between two SiPMs produced by KETEK (https://www.ketek.net/sipm/sipm-products/wb-series/, accessed on 31 July 2023) (models PM1125-WB and PM3325-WB), complemented by two detectors produced by HAMAMATSU Photonics (https://www.hamamatsu.com/eu/en/product/optical-sensors/mppc/mppc_mppc-array.html, accessed on 31 July) (models S13360-1350 and S13360-1375). The main characteristics of the SiPMs, as reported by their data sheets, are shown in Table 3. The SiPM under evaluation were selected on the base of the Photon Detection Efficiency (PDE) at 620 nm, the sensitive area, the Dark Count Rate (DCR) and the Optical Cross-Talk (OCT). In this study, reda comparison is made between two SiPMs produced by KETEK (https://www.ketek.net/sipm/sipm-products/wb-series/, accessed on 31 July 2023) (models PM1125-WB and PM3325-WB), complemented by two detectors produced by HAMAMATSU Photonics (https://www.hamamatsu.com/eu/en/product/optical-sensors/mppc/mppc_mppc-array.html, accessed on 31 July 2023) (models S13360-1350 and S13360-1375). The main characteristics of the SiPMs, as reported by their data sheets, are shown in Table 3. The SiPMs under evaluation were selected on the base of the Photon Detection Efficiency (PDE) at 620 nm, the sensitive area, the Dark Count Rate (DCR) and the Optical Cross-Talk (OCT).

**Figure 1:**
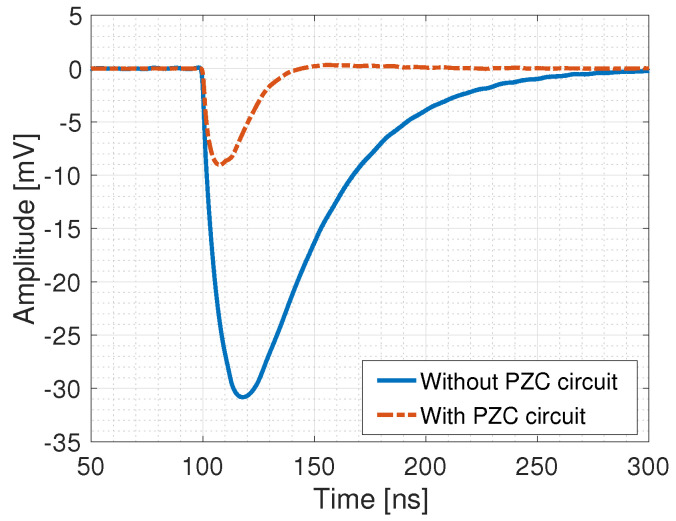
As an exemplary illustration showing the average signal of a single photo-electron signal obtained with the PM1125 SiPM before and after the PZC circuit. PZC reduces the signal duration from ∼150 ns to ∼35 ns.

**Figure 2:**
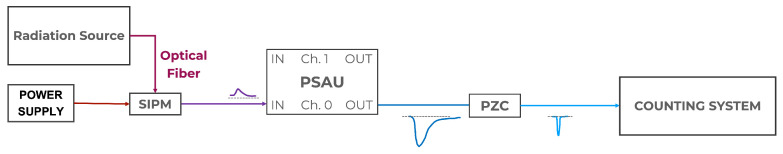
Schematic configuration of the experimental set-up.

The SiPMs were interfaced with a single PMMA optical fibre (0.5 mm diameter) with a scintillating YVO tip. In the prototype being commissioned, the signal by the SiPM was amplified (36 dB) by the CAEN SP5600 Power Supply and Amplification Unit (https://www.caen.it/products/sp5600e/, accessed on 31 July 2023), providing a single photo-electron (p.e.) signal peak amplitude in the 15–30 mV range, depending on the SiPM being considered.. To reduce the impact of piled-up events, the amplified signal was filtered by a detector-customised passive Pole-Zero Cancellation (PZC) circuit to reduce its duration (Figure 1). Finally, the signal was fed to a pulse counting unit to measure the rates. The schematic configuration of the system is shown in Figure 2.

o operate the system under stable and reproducible conditions and to avoid radiation protection issues, the characterisation was performed by irradiating the sensors with an X-ray beam, following the procedure outlined in [23]. The X-ray cabinet in use (model 554-81 by Leybold) emits photons in the 0–35 kVp range and provides currents up to 1 mA, tunable with a granularity of 0.01 mA. The X-ray flux produces rates up to a few MHz, comparable to what is expected in HDR brachytherapy. In order to guarantee the correct positioning of the fibre tip a dedicated “hose” was designed and machined; the clear fibre conveying the signal to the SiPM was routed through a cable duct to the detector. Photo-electron counting rates were measured using an Agilent Technologies DSO5054A oscilloscope, ((https://www.keysight.com/us/en/product/DSO5054A/5000-series-oscilloscope-500-mhz-4-channels.html, accessed on 31 July 2023) fed by the output signal of a CAEN 84 leading edge discriminator, operating on the PZC-shaped SiPM signal. The duration of the discriminator output pulse was set to 35 ns, matching the development of the analog signals.

The different SiPMs were compared based on the following criteria:

- **Minimum Detectable Rate (MDR)**, defined as the minimum Photon Counting Rate (PCR) exceeding 3 times the Poisson fluctuations of the DCR in the defined measurement time window:
  (2) $$\mathrm{MDR}\cdot \Delta t=3\sigma_{noise}\left(\Delta t\right)=3\cdot \sqrt{\mathrm{DCR}\cdot \Delta t}.$$
  This parameter is retained as a measurement of the minimum detectable dose rate above the DCR background. The time windows (Δt) are 0.1 s (HDR) and 0.5 s (LDR).
- **Linearity Range**, constrained by the MDR (lower limit) and event pile-up (upper limit). To obtain the measurements a current scan was performed from 0.05 mA to 1 mA in increments of 0.05 mA up to 0.3 mA and increments of 0.1 mA thereafter. To avoid systematic errors that could be introduced by the afterglow and the memory effect of the sensor [21], the DCR was measured after every irradiation and subtracted to obtain the PCR. The linearity range is measured by including measurements as frequencies increase until the ${\bar{\chi }}^{2}:=\chi^{2}/d.f.e$ exceeds 2.7 (corresponding to ≈90% Confidence Level), where $\chi^{2}$ is the sum of the normalised squared differences between measurements and predictions and *d*.*f*.*e*. is the number of degrees of freedom.
- **Sensitivity**, defined as the minimum variation of the X-ray current (I) that induces a change in PCR exceeding three standard deviations σ of a single measurement (ΔPCR≥3σ). In the linear regime, it may be written as
  (3) PCR=m·I,
  and the sensitivity is evaluated from the slope of the linear trend.

**Figure 3:**
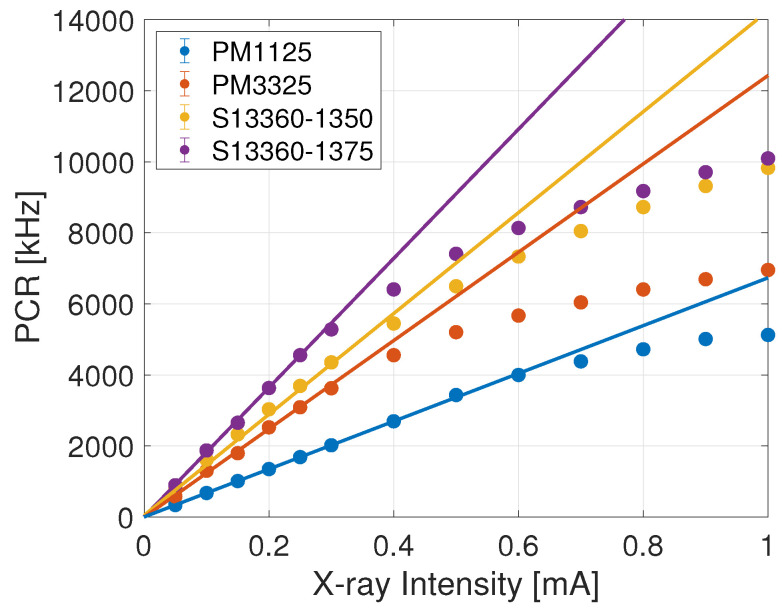
Response function of the SiPMs at 0.5 p.e. trigger threshold. The lines represent the linear fit. Points were excluded in the linear fit to have a ${\bar{\chi }}^{2}<2.7$.

Data were acquired by setting a threshold of 0.5 and 1.5 photo-electrons at the discriminator, namely with the comparator threshold set at half and three times half of the signal peak amplitude for the single-fired cell of the SiPM. Results for the 0.5 p.e. threshold are reported in Table 4 and Figure 3.

For LDR, the key parameters are the MDR and the Sensitivity, pointing to the use of the 75 μm pitch detector by HAMAMATSU, an expected result once the PDE is evaluated with respect to the S13360-1350, and considering the 69% larger sensitive area compared to the KETEK detectors.

**Figure 4:**
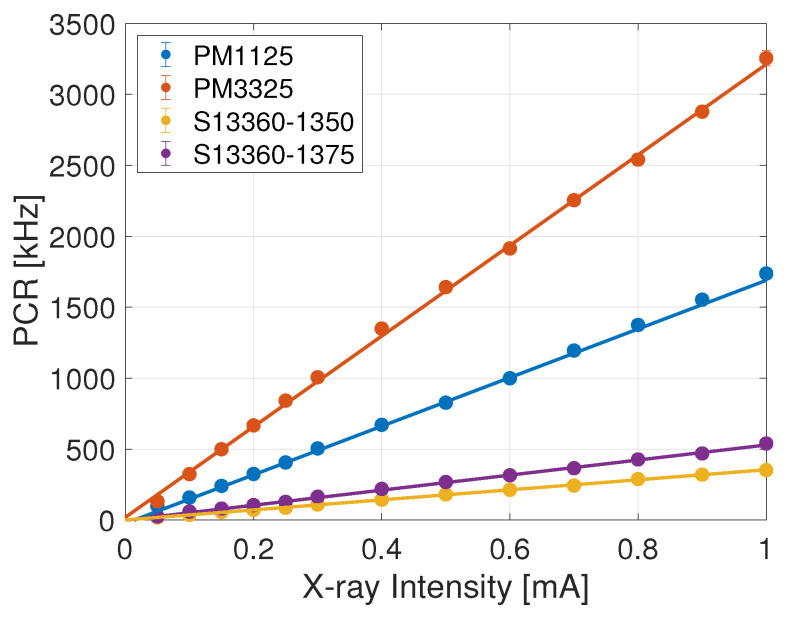
Response function of the SiPMs at 1.5 p.e. trigger threshold. The lines represent the linear fit.

When HDR is addressed, linearity is the key indicator and all the SiPMs are linear up to counting rates (PCR + DCR) of approximately 4 MHz. This could be a limiting factor for the closest distance with respect to the radioactive seed, where rates of up to 20 MHz are expected. In this respect, exploiting the SiPMs’ OCT offers a solution. In fact, linearity can be recovered while retaining an acceptable sensitivity by gauging the dose rates with respect to the counting rates at 1.5 p.e. threshold, thereby profiting from what is commonly considered a problematic feature of SiPMs. Results for the 1.5 p.e. threshold results are reported in Table 5 and Figure 4.

Measurements favour the KETEK SiPMs. In fact, even though linearity is recovered for all SiPMs over the full current range, the KETEK detectors have the highest sensitivity, a direct consequence of their OCT. PM3325 out performs PM1125 in terms of sensitivity, due to its larger geometrical acceptance, namely the covered solid angle with respect to the fibre tip; however, its higher DCR impacts the effective PCR linear range and MDR. Consequently, the optimal choice for the HDR SiPM falls to PM1125, with the caveat to verify whether its MDR and sensitivity permit achieving the required specifications as for Table 2.

## 4. Pre-Clinical Trial of the Prototype Dosimetric System

### 4.1. High Dose Rate Measurements

**Figure 5:**
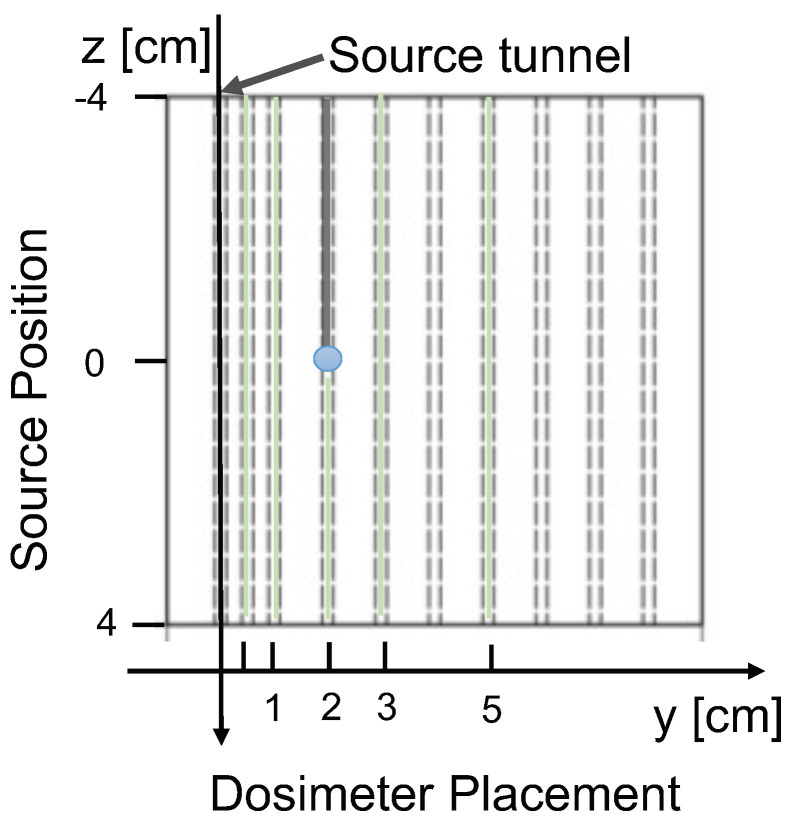
Drawing of the phantom used during the measurements. The blue dot identifies the position of the scintillating tip of the sensor.

A HDR measurement study was performed at Insubria University Hospital (Ospedale di Circolo Varese, Italy). The reported results were obtained with the PM1125 SiPM and the YVO fibre (0.5 mm diameter) irradiated by an ^192^Ir source of 4.84 Ci activity, with the radioactive core encapsulated in a steel capsule with length L = 3.6 mm and 0.65 mm diameter, delivered through a Nucletron microSelectron-HDR v2 afterloader. The assessment of the system under development was performed by inserting the source, and the sensor in a custom-made 1 cm thick water-equivalent phantom, with an area of 10 × 10 cm^2^. During the measurements, the phantom was immersed in a water tank with a volume of 80 × 80 × 80 cm^3^ to guarantee homogeneity in the surrounding material. A sketch of the phantom is illustrated in Figure 5. A series of tunnels were machined in the phantom; the source was positioned in the labelled tunnel and moved along the z-axis, while the sensor was positioned at different y distances, at z=0 cm. The tunnel diameters are 2.1 mm to allow the use of 6F catheters with 2 mm outer diameter and 1.5 mm internal diameter. Taking into account the source capsule external diameter of 0.9 mm, the distance to the sensor has a maximum positioning uncertainty of ±350μm. Sensors, jacketed in light protecting black tubing with 2 mm outer diameter, were moved within the phantom. The radioactive seed, embedded in the tip of a catheter, was moved along z in 2.5 mm increments when the sensor was at y=0.5 cm. For the remaining positions (1, 2, 3 and 5 cm in this data set), 2.5 mm increments were taken until |z|<2 cm and 5 mm for |z|>2 cm. The afterloader allowed for sub-mm repeatability on the source position, together with the possibility to program the sequence of positions as well as their respective dwell time.

The objectives of the measurement study were to test the compliance of the single-probe ORIGIN prototype with the TG43-U1 protocol and to assess its performance with respect to the project specifications.

**Figure 6:**
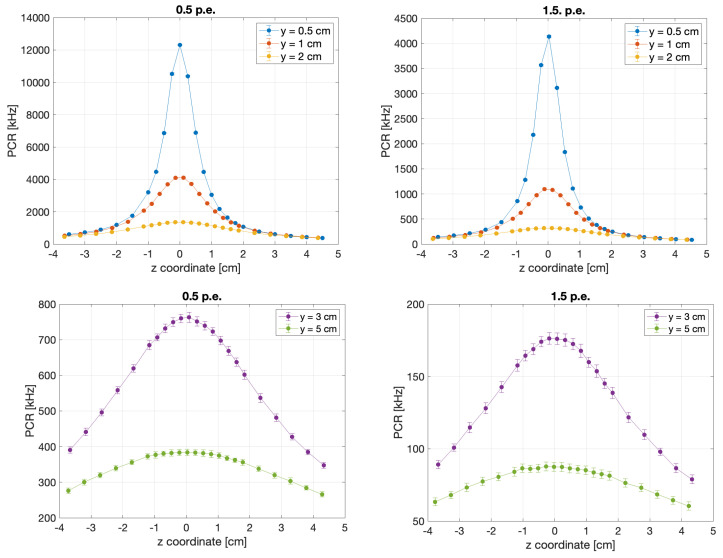
PCR curves obtained in the pre-clinical trials. The left panel shows the curves obtained with a threshold of 0.5 p.e. and those with a threshold of 1.5 p.e. are shown on the right For the sake of clarity, results for distances y = 0.5, 1 and 2 cm are shown on the top row while data for y = 3 and 5 cm are on the bottom row.

Figure 6 shows the measured curves for the PCR against the z-coordinate of the source. The measurements have been acquired with two detection thresholds (0.5 p.e., left panel, and 1.5 p.e., right panel) at different y-positions.

**Figure 7:**
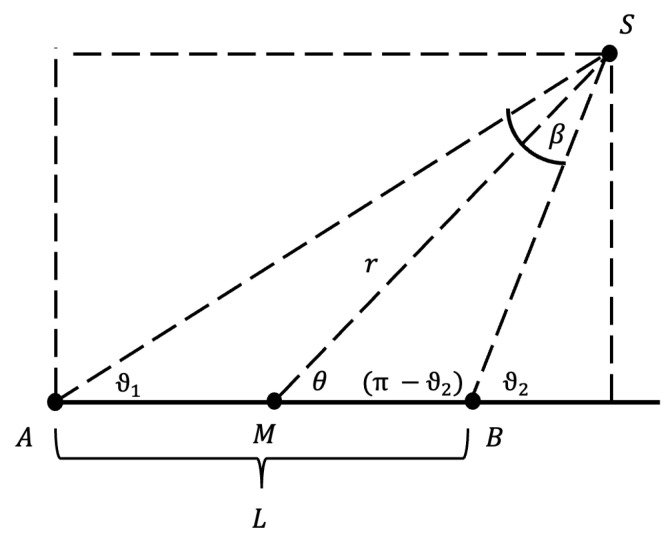
Geometry for the definition of G(r,θ). *r* is the radial distance from the centre point of the seed (M) to the point of interest (S), $\theta ,\theta_{1},\theta_{2}$ and β are the angles of interest, with the first being the azimuthal angle. A and B represent the endpoints of the source, and L is its total length.

To check the TG43-U1 compliance of the system, a comparison was made between the measured photon-counting rates and the theoretical expectation for the dose rates $\dot{D}\left(r,\theta \right)$. The latter, according to the TG43-U1 protocol, can be calculated as:

(4) $$\dot{D}\left(r,\theta \right)=s_{k}\Lambda F\left(r,\theta \right)\frac{G(r,\theta )}{G(r_{0},\theta_{0})}g\left(r\right),$$

where *r* is the distance to the source, θ is the azimuthal angle of the vector going from the sensor centre to the source centre, $r_{0}=2$ cm and $\theta_{0}=\pi /2$ are the normalization point, namely where data and prediction are gauged to get the conversion factor applied to all the complementary measurements. Normalisation was performed at 2 cm distance to ensure systematic errors due to both pulse pile-up and stem effect were not introduced. The parameters $s_{k}$ and Λ are two constants (the Air-Kerma strength and the dose rate constant) which depend on the radiation source. F(r,θ) is the anisotropy factor, G(r,θ) is the geometry factor and g(r) is the radial dose function [15]. The anisotropy factor takes into consideration the geometry of the capsule containing the source and the interactions of the radiation with it; its value is retrieved from look-up tables (https://physics.carleton.ca/clrp/seed_database/, accessed on 31 July 2023), and it does not have an analytical expression. The geometry factor models the geometrical aspect of the source and is given by

(5) $$G\left(r,\theta \right)=\{\begin{array}{cc} \frac{\beta }{Lr\sin\left(\theta \right)} & \mathrm{if}\theta \neq 0^{\circ },\\ {\left(r^{2}-L^{2}/4\right)}^{-1} & \mathrm{if}\theta =0^{\circ }, \end{array}$$

where $\beta =\theta_{2}-\theta_{1}$ is defined as in Figure 7 and *L* is the length of the cylindrical source.

Finally, the radial dose function accounts for the interaction of the radiation in the water-equivalent material and for a cylindrical source, it is defined by the heuristic expression [24]

(6) $$g\left(r\right)=\left(\sum_{i=-2}^{3}a_{n}r^{n}\right)e^{-a_{e}r},$$

where $a_{n}$ are coefficients determined by the type of source and afterloader used for the treatment. It is thus important to remark that all the terms in (4) depend on the source and afterloader used.

**Figure 8:**
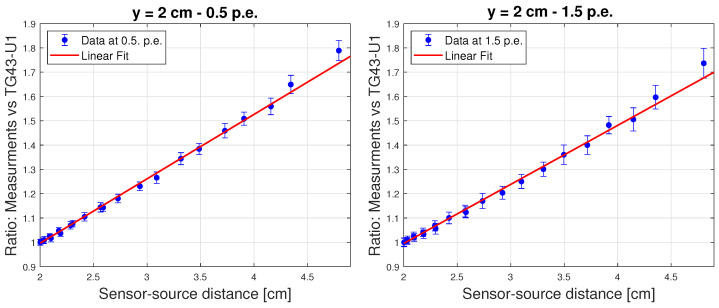
Energy dependence correction curves for the sensor placed at a 2 cm distance from the source tunnel. The left panel shows the result obtained for 0.5 p.e. detection threshold and the right panel the one for a 1.5. p.e.. The error bars represent the spread of the underlying distribution.

The non-water equivalence of the scintillator was studied through the ratios between the normalised experimental data and the corresponding theoretical predictions versus the source-sensor distance, obtaining the energy correction curves. Exemplary results for the sensor positioned at 2 cm from the source tunnel for both counting thresholds are shown in Figure 8, featuring the expected linear trend and confirming TG43-U1 compliance [20,21,25] at the reported distance.

**Figure 9:**
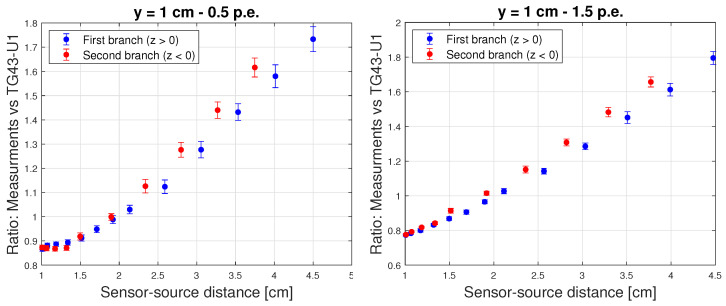
Energy dependence correction curves for the sensor placed at a 1 cm distance from the source tunnel. The left panel shows the result obtained for 0.5 p.e. of detection threshold, and the right panel the one for a 1.5. p.e.. The error bars represent the spread of the underlying distribution.

However, for the plots obtained with the sensor closer to the source tunnel (y = 1 cm), the curves display a clear non-linear trend (Figure 9), resulting from the superposition of two effects: piled-up events and the “stem effect”. The latter is associated with two processes: Cherenkov light produced by the impinging radiation on the fibre and photoluminescence induced by the same Cherenkov light on the scintillating material [20,21]. The impact of piled-up events is clearly visible comparing the left and right panels of Figure 9, where the measurements at 0.5 p.e. and 1.5 p.e. are shown. At the higher threshold, where the counting rates are smaller by about a factor 3, linearity at the smallest source-sensor distances is almost fully recovered. However, even at 1.5 p.e. threshold, some degree of non-linearity persists and notably the energy dependence curves splits into two branches, due to the “stem effect”. In fact, since the acceptance of the fibre to the radiation field does depend on the source position along z, the impact of Cherenkov radiation induced effects is expected to be asymmetrical with respect to z = 0, with a larger effect at negative z values (Figure 5). Nevertheless, the use of Cherenkov light filters positioned in front of the detector and possibly in front of the scintillating material was shown to solve the issue in [20,21,26] and it will also be implemented for the ORIGIN system, targeting the recovery of TG43-U1 compliance over the full range of y-values.

**Figure 10:**
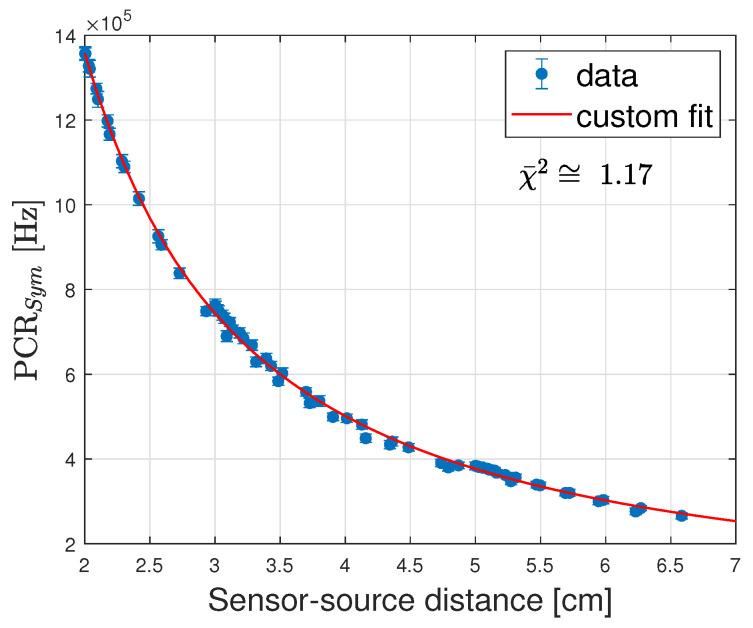
Fit of the ${PCR}_{Sym}$ curve obtained from the merger of the data at all the y-values for which the stem effect is negligible as a function of the distance. We obtain ${\bar{\chi }}^{2}≅1.17$.

To evaluate the performance of the prototype under study in HDR brachytherapy with respect to the figures specified in Table 2, the angular dependence needs to be decoupled from the source-sensor distance in the PCR and the analysis needs to be limited to data with negligible stem effect (y values ≥2 cm), obtained at 1.5 p.e. counting threshold. As described in Appendix A, this is possible by approximating the geometry factor and dividing the recorded measurement by a calculated θ-dependent anisotropy factor, to yield what is named here as ${PCR}_{Sym}\left(r\right)$, to be compared to the radially dependent function

(7) $$h\left(r\right)=\left(\frac{a}{r^{2}}+\frac{b}{r}+c\right)e^{-dr},$$

where *a*, *b*, *c* and *d* are free parameters. The result is reported in Figure 10.

Since the model function fits the data well, the fit can be reasonably relied upon to extrapolate the ${PCR}_{Sym}$ trend at 10 cm (where de facto, coincides with the PCR), for a value of 27±3 kHz, well in excess of the required specifications (MDR of 1.5±0.1 kHz as of Table 4). Furthermore, by relying on the verified Poissonian hypothesis, the Statistical Precision (1) may be written as

(8) $$SP\left(r;\Delta t\right)={\frac{\sqrt{N_{PCR}\left(r\right)+N_{DCR}}}{N_{PCR}\left(r\right)}}_{|\Delta t},$$

where $N_{DCR}$ is the expected distance-independent DCR count in the selected time window, and $N_{PCR}$ is the PCR count in the specified measurement time interval Δt. Using (8) a value is obtained for the statistical precision in 0.1 s at 10 cm of 2.95±0.08 %, acceptably within the specification (5%). Finally, a straightforward way to evaluate the spatial resolution is through the first derivative of ${PCR}_{Sym}\left(r\right)$

(9) $$\Delta R\left(r\right)={\left[3\sigma_{PCR}\cdot |{\left(\frac{\partial PCR}{\partial r}\right)}^{-1}|\right]}_{|r}.$$

Using the fit function shown in Figure 10 and (9), a spatial resolution of 0.40±0.03 mm is obtained at 5 cm with respect to the requested value of 1 mm. Hence, it can be concluded that the ORIGIN prototype, with the SiPM selected for HDR, meets all the specifications requested by the project, assuming that the Cherenkow filters reduce the stem effect for y-distances ≤1 cm.

### 4.2. Pre-Clinical Campaign: LDR

**Figure 11:**
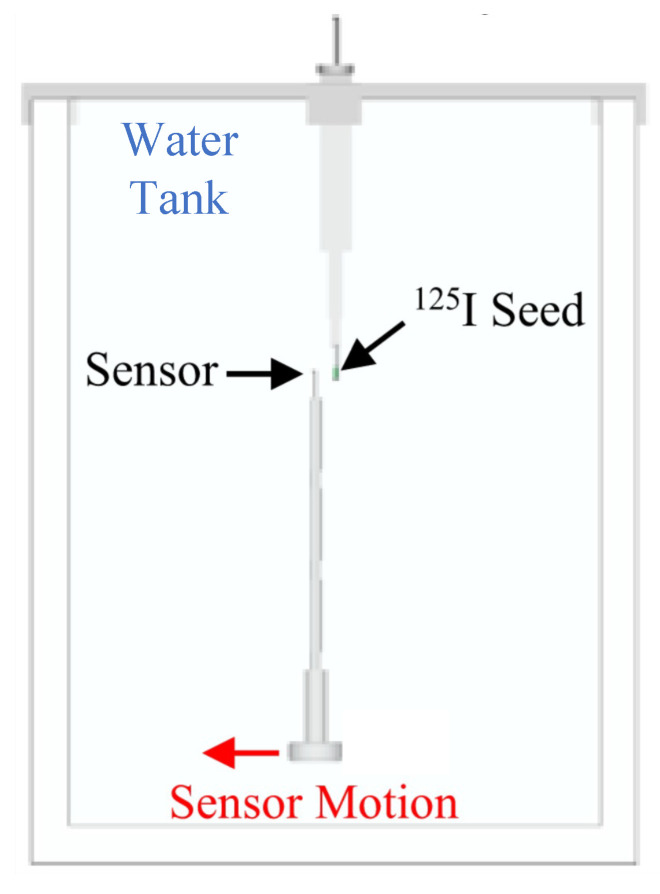
Schematic view of water phantom setup employed in LDR measurements [27].

**Figure 12:**
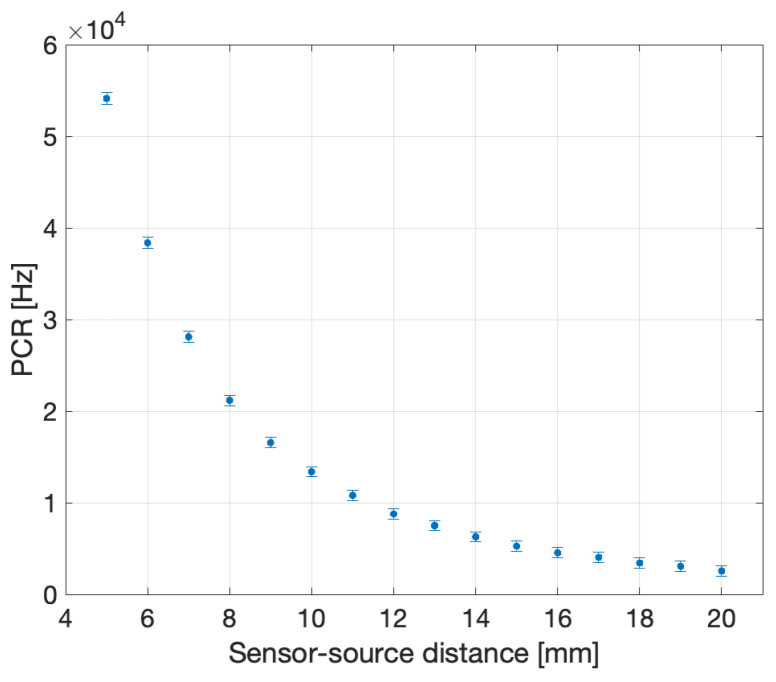
PCR curve obtained in the pre-clinical trial.

An LDR measurement study was performed at the Radiotherapy Department of the Galway Clinic (Galway, Ireland). The results were obtained with the S13360-1375 SiPM and the Gadox fibre (0.5 mm diameter) irradiated by an ^125^I source of 0.3 mCi activity. The measurements were carried out using a PTW MP3-XS water phantom system (PTW, Freiburg, Germany), coupled with a 3-D printed source and sensor holders made from polymer-like material with a density of 1.17–1.18 g/cm^3^ [27]. Figure 11 shows the schematic of the setup. The ^125^I radioactive seed was introduced into the water tank, aligned with respect to the fibre dosimeter, and then moved along the radial direction with steps of 1 mm (precision of 0.1 mm) from 5 mm up to 20 mm. DCR runs were taken and subtracted to all the measurements to obtain the PCR. Figure 12 shows the PCR as a function of the sensor-source distance.

**Figure 13:**
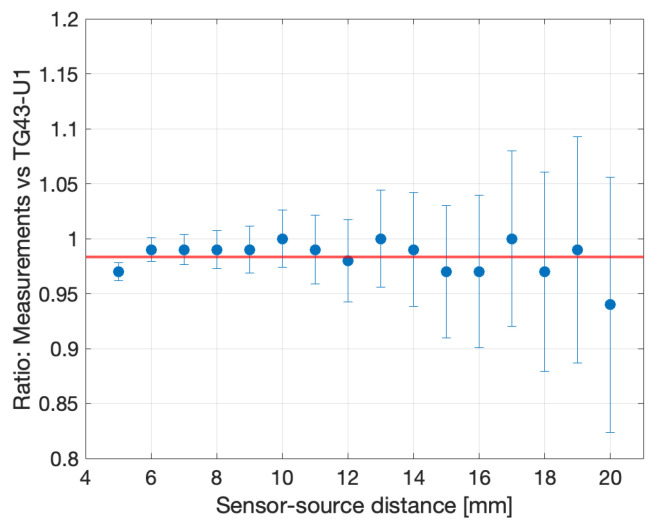
Ratios between the measured PCR and the TG41-U1 prediction. The error bars represent the spread of the underlying distribution.

Because of the low-energy radiation typically emitted by LDR sources, energy correction is not expected to be required [27] and the data support this, as shown in Figure 13, where the ratios between the dose-rate-normalized PCR and the TG43-U1 predictions are displayed.

**Figure 14:**
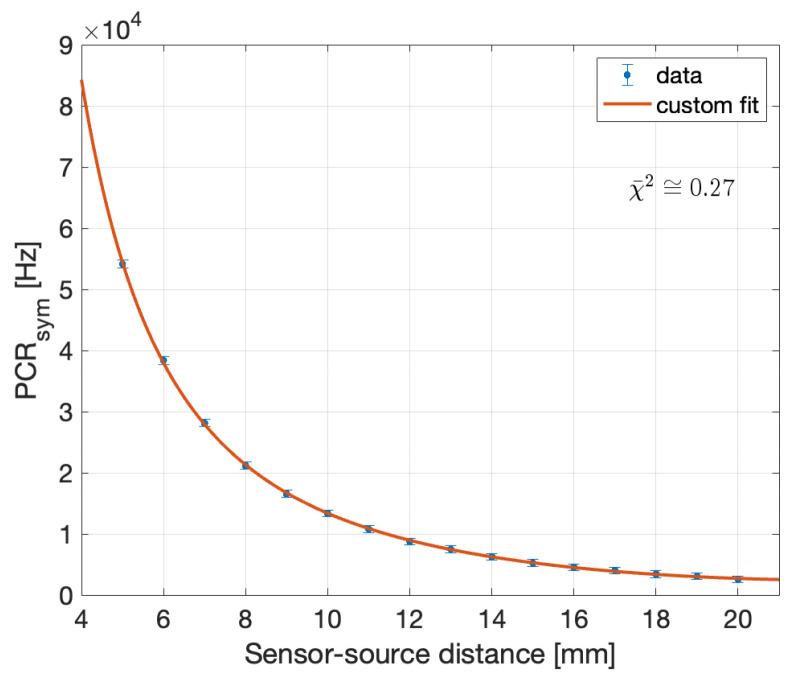
Fit of the PCR as a function of the distance.

Assessment of the system performance against the project specification was pursued following the approach outlined for HDR dosimetry, with the advantage that angular dependence did not need to be considered since all the data were recorded on the plane perpendicular to the source centre. Moreover, the seed longitudinal dimension is 3.5 mm, and the geometry factor differs from that of a point-like source by less than 5% already at r=7 mm and quickly reaches levels below 1%. Therefore, the radial dependence of the PCR was fitted to the curve used in HDR brachytherapy, as shown in Figure 14.

The recorded PCR exceeds the required 1.2 kHz MDR for distances below 2.5±0.1 cm, actually shorter than the maximum 3 cm of clinical interest. The statistical precision falls below the 5% in 0.5 s at a distance of 1.34±0.1 cm short of the intended target of 3 cm. Finally, the spatial resolution drops under 3 mm at a distance of 2.2±0.1 cm.

## 5. Conclusions

The ORIGIN project aims to develop an in-vivo real-time dosimeter for oncological brachytherapy, with source-tracking and dose-mapping capabilities.

The proposed solution, based on clear fibres with a scintillator embedded in the tip and a decay time in the order of 500 μs, emitting a series of single photons detected by SiPM, has been designed and commissioned. A series of SiPM were selected and tested in laboratory conditions to optimise the system performance with respect to minimum detectable rate, linearity range and sensitivity, with different specifications for Low Dose Rate (LDR) and High Dose Rate (HDR) treatments.

Two single-probe prototypes of the ORIGIN system were tested with actual brachytherapy sources for HDR and LDR treatments. For HDR, the system was proven to be TG43-U1 compliant when stem related effects are negligible, namely at distances in excess of 2 cm; solutions based on fibre splitting and filtering are currently being investigated to overcome these limitations. For LDR, comparison to tabulated data was limited to radial distance variations. In HDR brachytherapy, the system has been proven to meet and exceed the design specifications. On the other hand, for LDR, its sensitivity was found to be limited by the Dark Count Rate values. In order to overcome this issue, the use of thermo-electric cooled SiPMs together with a custom optical system for avoiding potential limitations due to the T0-8 package is envisaged, which is expected to reduce the DCR by approximately one order of magnitude when operated at −20 °C.

It is worth noting that:

- The accuracy of the point-to-point DCR subtraction is confirmed by the value of the offset in the linear fit, statistically compatible with zero;
- The difference in the sensitivity between the two HAMAMATSU SiPM can be traced to the different Photon Detection Efficiency. In fact, the ratio between the two values is 1.28±0.04, compatible with the PDE ratio (1.25, as of the data sheet and Table 2).
- The upper limit in the linearity range, once the DCR is accounted for, corresponds for all the detectors to a counting rate of ≈4 MHz (corresponding to a pile-up slightly above 10%), as expected by the comparable development time after the PZC and the discriminator logical signal duration.

## Figures and Tables

**Table 2:** Specification of the ORIGIN project, for HDR and LDR.

	LDR	HDR
Sensitivity to Dose	up to 3 cm	up to 10 cm
Spatial Resolution	3 mm @ 3 cm	1 mm @ 5 cm
Dose Rate Range	0.4–2 Gy/h	>12 Gy/h
Statistical Precision	5% in 0.5 s @ 3 cm	5% in 0.1 s @10 cm

**Table 3:** Main characteristics of PM1125, PM3325, S13360-1350, and S13360-1375. DCR and Breakdown Voltage are referred to a 25 °C temperature.

	PM1125	PM3325	S13360-1350	S13360-1375
Sensitive area [mm^2^]	1 × 1	3 × 3	1.3 × 1.3	1.3 × 1.3
Cell pitch μm	25	25	50	75
PDE at 420 nm	45%	45%	40%	50%
PDE at 620 nm	17%	17%	24%	30%
DCR [kHz]@ 0.5 p.e.	125	1125	90	90
Optical cross-talk	26%	26%	3%	7%
Breakdown Voltage $V_{bd}$ [V]	24	24	53	53
Operational Voltage [V]	$V_{bd}$ + 5	$V_{bd}$ + 5	$V_{bd}$ + 3	$V_{bd}$ + 3
Gain at operational voltage	1.7 $\times \;10^{6}$	1.7 $\times \;10^{6}$	1.7 $\times \;10^{6}$	4.0 $\times \;10^{6}$

**Table 4:** Values for the key indicators of the detectors at a detection threshold of 0.5 p.e.

	PM1125	PM3325	S13360-1350	S13360-1375
MDR in 0.1 s [kHz]	3.1 ± 0.2	10.5 ± 0.3	3.0 ± 0.2	2.8 ± 0.2
MDR in 0.5 s [kHz]	1.4 ± 0.05	4.7 ± 0.1	1.4 ± 0.05	1.2 ± 0.05
Linearity upper limit (PCR)	0.6 mA	0.25 mA	0.3 mA	0.25 mA
	4.04 MHz	3.09 MHz	4.31 MHz	4.43 MHz
Sensitivity (m) [kHz/mA]	6710 ± 31	12,438 ± 448	14,203 ± 354	18,187 ± 378

**Table 5:** Values for the key indicators of the detectors under test for a detection threshold of 1.5 p.e.

	PM1125	PM3325	S13360-1350	S13360-1375
MDR in 0.1 s [kHz]	1.5 ± 0.1	5.5 ± 0.2	0.49 ± 0.07	0.47 ± 0.07
MDR in 0.5 s [kHz]	0.68 ± 0.04	2.5 ± 0.07	0.22 ± 0.002	0.21 ± 0.002
Linearity upper limit (PCR)	1 mA	1 mA	1 mA	1 mA
Sensitivity (m) [kHz/mA]	1713 ± 22	3192 ± 31	354 ± 3	533 ± 4

## Data Availability

Data available on request.

## Appendix A

As discussed in Section 4, the dependence of the dose measurements on the sensor-source distance and azimuthal angle may be written as:

(A1) $$PCR\left(r,\theta \right)=f\left(r\right)\dot{D}\left(r,\theta \right),$$

where the linearly dependent energy form factor (non-water equivalence) f(r) is explicitly made and $\dot{D}\left(r,\theta \right)$ is the prediction by the TG43-U1 protocol, that is

(A2) $$\dot{D}\left(r,\theta \right)=CF\left(r,\theta \right)G\left(r,\theta \right)g\left(r\right),$$

where *C* is a global constant, F(r,θ) is the anisotropy factor, G(r,θ) is the geometry factor and g(r) is the radial dose function.

An effective dependence on the radial distance alone may be obtained when ϵ=L/r≪1, which is the range of interest when calculations are restricted to data with negligible stem effect, namely r > 20 mm for L = 3.8 mm. Since $\theta_{1,2}$ may be written as (see Figure 7):

(A3) $$\begin{array}{c} \theta_{1}=\arcsin\left(\frac{r\sin\theta }{\sqrt{r^{2}-Lr\cos\theta +L^{2}/4}}\right)=\arcsin\left(\frac{\sin\theta }{\sqrt{1-\epsilon \cos\theta +\epsilon^{2}/4}}\right), \end{array}$$

(A4) $$\begin{array}{c} \theta_{2}=\arcsin\left(\frac{r\sin\theta }{\sqrt{r^{2}+Lr\cos\theta +L^{2}/4}}\right)=\arcsin\left(\frac{\sin\theta }{\sqrt{1+\epsilon \cos\theta +\epsilon^{2}/4}}\right). \end{array}$$

A second order Taylor expansion of the geometry factor results in the form

(A5) $$\begin{array}{cc} G(r,\theta ) & =\frac{\beta }{Lr\sin\theta }=\frac{\theta_{2}-\theta_{1}}{Lr\sin\theta }\\ \; & =\frac{\sum_{\pm }\arcsin\left[\sin\theta \left(1\pm \frac{\epsilon }{2}\cos\theta -\frac{\epsilon^{2}}{4}+\frac{\epsilon^{2}}{8r^{2}}\cos^{2}\theta \right)+o\left(\epsilon^{3}\right)\right]\mathrm{sgn}\left(\pm \right)}{Lr\sin\theta }\\ \; & =\frac{\epsilon \sin\theta +o\left(\epsilon^{3}\right)}{Lr\sin\theta }=\frac{1}{r^{2}}+o\left(\epsilon^{3}\right), \end{array}$$

As a consequence, in the range of interest, the geometry factor can essentially be approximated by what is expected for a point-like source, namely:

(A6) $$\tilde{G}\left(r\right)=\frac{1}{r^{2}},$$

Once the anisotropy factor is considered, it is worth reminding that in the region of interest the radial dependence accounts for variations at the 0.01% while the angular dependence brings about changes at the 0.1–1% level. Being so, it is possible to write F(r,θ)≈1+h(θ)=:F(θ), where h(θ) is a first-order correction. This is directly obtained from the values of F(r,θ) as reported by the look-up tables in https://physics.carleton.ca/clrp/seed_database/ accessed on 31 July 2023).

Lastly, within the boundaries of the domain of interest, a new quantity, $PCR_{Sym}\left(r\right)$, can be directly considered, given by

(A7) $$PCR_{Sym}\left(r\right):=\frac{PCR(r,\theta )}{F(\theta )}.$$

Namely, by referring to (A1), (A2), (6), (A6) and (A7):

(A8) $$PCR_{Sym}\left(r\right)=C\frac{f(r)}{r^{2}}\left(\sum_{n=-2}^{3}a_{n}r^{n}\right)e^{-a_{e}r}=\left(\sum_{n=-4}^{2}c_{n}r^{n}\right)e^{-a_{e}r}.$$

Hence, $PCR_{Sym}\left(r\right)$ could be modeled using the r.h.s. of (A8). However, a further step can be taken if we consider that the theoretical function would need an eight-parameter fit. Unnecessary components in (A8), which end up adding noise rather than information for the fit in the region of interest, should be eliminated. The coefficients related to the fourth and third power of 1/r can be readily discarded as they decay much faster than the others; thus, it is expected they will be completely negligible in determining the quantities of interest at large distances. The $1/r^{2}$ term represents the simple geometric dilution of the radiation, and the constant term can be related to stable background radiation. All the other components are ascribable to modelling the complex interaction of the radiation with the medium (absorption, scattering etc.). In particular, the exponential term is introduced in order to regularize the function at large *r*. On the other hand, considering the positive powers of *r*, these terms could be neglected only if their coefficients were to be of many orders of magnitudes smaller than the ones for the negative powers (to compensate for the growth of importance of these terms with the distance). If it was so, these terms could be removed without hindering the explanatory power of the model. Indeed, by performing a fit of the data for $PCR_{Sym}\left(r\right)$, both for the HDR and LDR data, using the full theoretical expression. In this case, the reader must be reminded that the correct expression to use for the fit in LDR is not (A8), but it is given by $PCR_{Sym}=\left(\sum_{n=-4}^{1}c_{n}r^{n}\right)e^{-a_{e}r}$, as f(r)=const in LDR. It can be found that $c_{n\geq 1}/c_{n\leq 0}\approx 10^{-5}$ and, moreover, the coefficients $c_{-4}$ and $c_{-3}$ are one order of magnitude smaller than those of the other negative powers thus making these terms uninfluential at large distances. Hence, for $PCR_{Sym}\left(r\right)$, the simpler model can be employed

(A9) $$PCR_{Sym}\left(r\right)=\left(\frac{a}{r^{2}}+\frac{b}{r}+c\right)e^{-a_{e}r}.$$
